# Engineered Bacteria for Disease Diagnosis and Treatment Using Synthetic Biology

**DOI:** 10.1111/1751-7915.70080

**Published:** 2025-01-13

**Authors:** Kai Jin, Yi Huang, Hailong Che, Yihan Wu

**Affiliations:** ^1^ Department of Environmental and Chemical Engineering Shanghai University Shanghai China

**Keywords:** biosensor, engineered bacteria, live biotherapy, synthetic biology

## Abstract

Using synthetic biology techniques, bacteria have been engineered to serve as microrobots for diagnosing diseases and delivering treatments. These engineered bacteria can be used individually or in combination as microbial consortia. The components within these consortia complement each other, enhancing diagnostic accuracy and providing synergistic effects that improve treatment efficacy. The application of microbial therapies in cancer, intestinal diseases, and metabolic disorders underscores their significant potential. The impact of these therapies on the host's native microbiota is crucial, as engineered microbes can modulate and interact with the host's microbial environment, influencing treatment outcomes and overall health. Despite numerous advancements, challenges remain. These include ensuring the long‐term survival and safety of bacteria, developing new chassis microbes and gene editing techniques for non‐model strains, minimising potential toxicity, and understanding bacterial interactions with the host microbiota. This mini‐review examines the current state of engineered bacteria and microbial consortia in disease diagnosis and treatment, highlighting advancements, challenges, and future directions in this promising field.

## Graphical abstract

Bacteria including probiotics, genetically engineered bacteria, and microbial consortia are employed in disease diagnosis and treatment via diverse mechanisms.

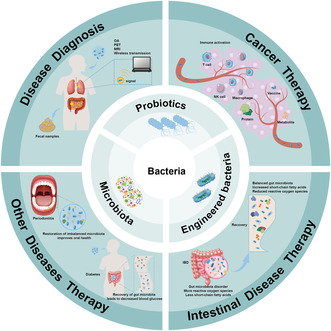

## 1 Introduction

William Coley's earliest attempts to use inactivated bacteria (Coley's toxin) to prime the immune system to fight tumours became an early cornerstone of cancer immunotherapy (Chakrabarty 2003; Karbach et al. 2012). Despite the limited technology in the early days, with advances in immunology and molecular biology, the potential of bacterial therapies was re‐emphasised with clinical results, one of the most successful examples being the FDA approval of *Mycobacterium bovis* Bacillus Calmette‐Guérin (BCG) vaccine for the treatment of bladder cancer (Guallar‐Garrido and Julián 2020; Lange et al. 2022). Today, genetic engineering has enabled bacteria to become precision medicine tools, modified to not only detect disease biomarkers and provide early diagnosis but also to produce anticancer drugs or immunomodulators to improve therapeutic efficacy.

**FIGURE 1:**
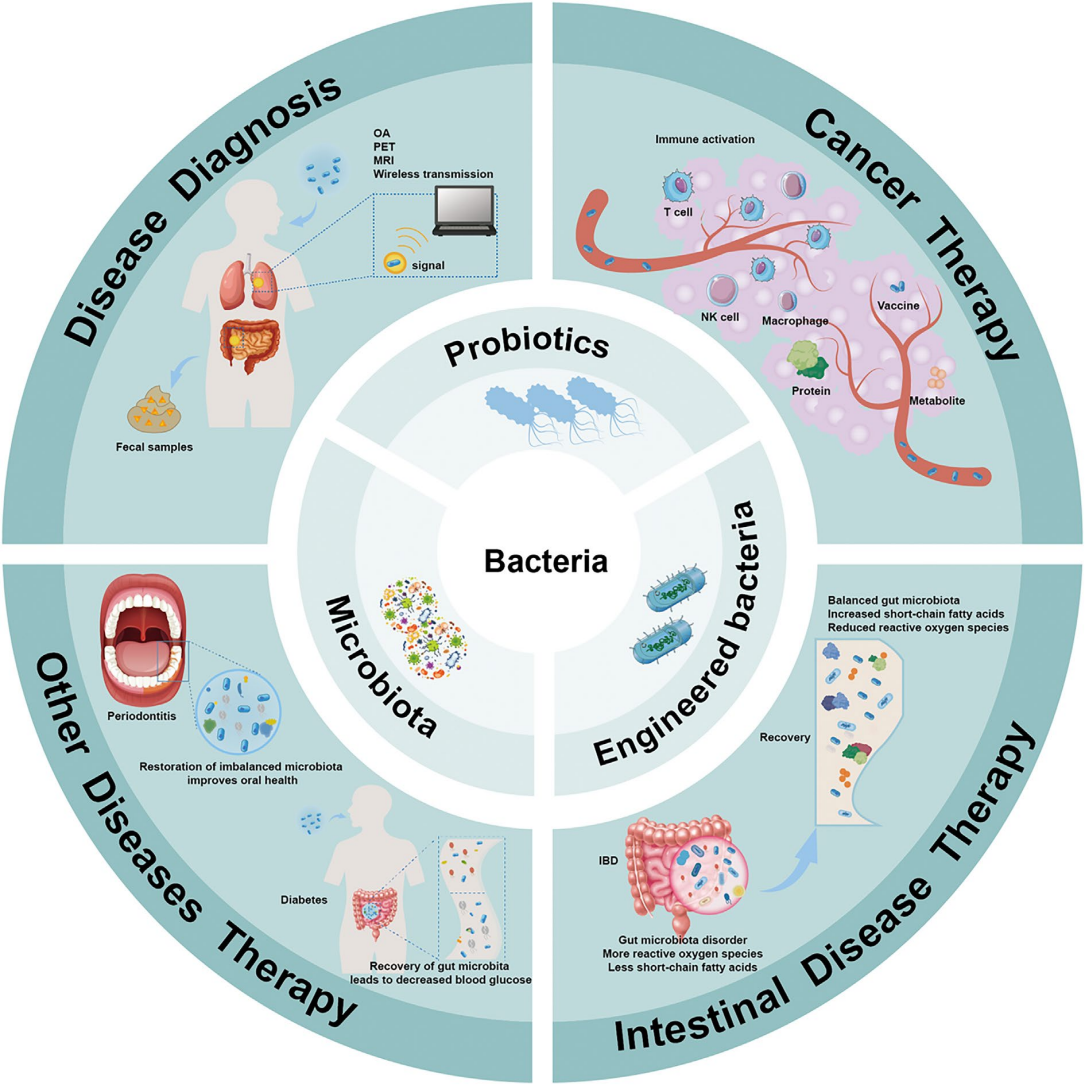
Bacteria in disease diagnosis and therapy. This figure illustrates the diverse roles of bacteria in disease diagnosis and treatment. Probiotics, genetically engineered bacteria, and the native microbiota contribute significantly to healthcare. Engineered bacteria can produce therapeutic proteins or metabolites, act as tumour vaccines, and activate immune responses, providing innovative cancer treatments. In the gastrointestinal tract, bacteria secrete beneficial compounds such as short‐chain fatty acids, restoring healthy microbiota and treating intestinal diseases. Furthermore, bacteria can alleviate conditions such as periodontitis and diabetes by modulating the human microbiota. Additionally, bacteria function as biosensors, detecting diseases through various signals. These signals can be captured using optoacoustic imaging (OA), positron emission tomography (PET), magnetic resonance imaging (MRI), and wireless transmission or by analysing faecal samples.

Recent advances in bacterial engineering technologies have introduced promising new approaches for disease diagnosis and treatment (Zhou 2016). Using recombinant and metabolic engineering techniques, researchers have developed bacteria capable of detecting disease‐specific biomarkers, facilitating non‐invasive, real‐time diagnostic applications (Tanniche and Behkam 2023). With CRISPR technology, engineered bacteria can now recognise specific nucleic acids, such as those involved in cancer mutations or viral infections (Cooper et al. 2023). For therapeutic purposes, bacteria can be directed to disease sites to deliver therapeutic agents precisely; for instance, tumour‐targeting bacteria can release drugs in a targeted manner through quorum‐sensing systems (Fan et al. 2022). Innovations in synthetic gene circuits have also enhanced the safety and controllability of bacterial therapies through self‐destruct mechanisms and external control switches (Gurbatri, Arpaia, and Danino 2022; Chiang and Hasty 2023). Delivery methods like hydrogel encapsulation (Yu et al. 2022) of engineered probiotics have demonstrated potential efficacy in treating gastrointestinal diseases and other pathological conditions (Li et al. 2021; Jiang et al. 2022). Nonetheless, bacterial therapeutics face challenges, including pathogenicity concerns, and undesirable immune reactions and interactions with host microbiota. This mini‐review summarises recent advances in bacterial engineering technologies for diagnosis and therapies (see Figure 1, Table 1) and discusses strategies to mitigate potential adverse effects of bacterial therapies and their broader implications for health.

**TABLE 1:** Examples of applications of genetically engineered bacteria in disease diagnosis and treatment.

Application	Engineered bacteria	Gene modification site	Disease model	Mechanism	Whether the strain/plasmid is publicly available	References
Diagnosis	*Acinetobacter baylyi*	Genome	Mice with colorectal cancer	Insertion of KRAS homology arms for homologous recombination with cancer DNA	Yes	Cooper et al. (2023)
	*Lactococcus lactis*	Genome	HEK293, HT‐29 and Caco‐2 cells	Expressing proteins binding the cancer‐related transmembrane receptors EpCAM and HER2	Yes	Plavec et al. (2021)
	*Escherichia coli* MG1655	Plasmid	Mice with colorectal cancer	Expressing tyrosinase and its “caddie” protein to produce melanin for contrast‐enhanced OA imaging	Yes	Yun et al. (2021)
	*Escherichia coli* Nissle 1917	Plasmid	Mice with colorectal cancer, lung cancer and pancreatic	Expressing β‐galactosidase which uses LuGal as the substrate to produce luminescence	Yes	Danino et al. (2015)
	*Escherichia coli* Nissle 1917	Plasmid	Mice with IBD	Expressing a nitrate‐dependent two‐component system to sense and respond to nitrate, enabling the detection of intestinal inflammation	Yes	Woo et al. (2020)
	*Escherichia coli* Nissle 1917	Plasmid	Mice with IBD	Expressing GFP under the regulation of NO‐sensitive ytfE and hmp promoters, enabling the fluorescence‐based detection of NO	Yes	McKay et al. (2018)
	*Escherichia coli* Nissle 1917	Plasmid	Mice with IBD	Expressing fluorescent protein under the regulation of ThsS/R which responds to thiosulfate	Yes	Zou et al. (2023)
	*Escherichia coli* MG1655	Genome	*Vibrio cholerae* infection in vitro	Expressing a gene circuit comprising CqsS, LuxU, and LuxO as sensors to regulate the production GFP as the reporter, enabling detection of CAI‐1 from *V. cholerae*	Yes	Holowko et al. (2016)
Therapy	NiCo21(DE3) *E. coli*	Plasmid	Mice with lymphoma and breast cancer	Releasing anti‐CD47 nanobody after controlled lysis regulated by quorum sensing	Yes	Chowdhury et al. (2019)
	*Bifidobacterium Infantis*	Plasmid	Mice with lung cancer	Expressing sFlt‐1 gene to inhibit VEGF‐induced tumour growth	Yes	Zhu et al. (2011)
	*Escherichia coli* Nissle 1917	Plasmid	Mice with melanoma	Expressing an anti‐angiogenic active fragment of tumstatin to inhibit angiogenesis	Yes	He et al. (2017)
	*Escherichia coli* Nissle 1917	Genome	Mice with colorectal cancer	Deleting the arginine repressor gene *argR* and expressing a feedback‐resistant *argA* to improve arginine production, which enhances the response of PD‐L1 blocking antibodies	Yes	Canale et al. (2021)
	*Lactococcus lactis*	Plasmid	Mice with colorectal cancer, breast cancer and melanoma	Expressing a fusion protein combining Fms‐like tyrosine kinase 3 ligand and OX40 ligand to modulate anti‐tumour immune responses	Yes	Zhu et al. (2022)
	*Staphylococcus epidermidis* NIHLM087	Plasmid	Mice with melanoma	Expressing melanoma tumour antigens for induction of highly specific T cell responses	Yes	Chen et al. (2023)
	*Escherichia coli* Nissle 1917	Plasmid	Mice with IBD	Expressing immunomodulator under the regulation of ThsS/R which responds to thiosulfate	Yes	Zou et al. (2023)
	*Escherichia coli* Nissle 1917	Plasmid	Mice with IBD	Expressing catalase and superoxide dismutase to reduce ROS for the treatment of intestinal inflammation	Yes	Zhou et al. (2022)
	*Escherichia coli* Nissle 1917	Plasmid	Mice with IBD	Expressing modified curli nanofiber matrix with anti‐inflammatory domains to promote in situ intestinal epithelial integrity	Yes	Praveschotinunt et al. (2019)
	*Escherichia coli* Nissle 1917	Plasmid	Mice with IBD	Sustainable production of 3‐hydroxybutyrate for the treatment of colitis	Yes	Yan et al. (2021)
	*Lactobacillus gasseri*	Genome	Mice with diabetes	Expressing GLP‐1 to induce differentiation of intestinal epithelial cells into functional glucose‐responsive insulin‐producing cells	Yes	Duan, Liu, and March (2015)

## 2 Bacteria for Diagnostic and Therapeutic Applications in Oncology

### 2.1 Bacteria for Oncological Diagnostics

The ability of bacteria to selectively colonise tumours helps them to be developed as programmable diagnostic devices. The advancement of synthetic biology offers novel prospects for creating in vivo sensors with enhanced sensitivity and diagnostic precision. For instance, *Acinetobacter baylyi* , renowned for its inherent capacity for horizontal gene transfer, integrates exogenous genetic material from the environment into its genome (Cooper, Tsimring, and Hasty 2017). Using CRISPR‐Cas9 technology, the homology arm of the human colorectal cancer gene KRAS was integrated into *A. baylyi* (Cooper et al. 2023). Concurrently, the tetracycline repressor (*tetR*) gene was inserted between the KRAS homology arms to inhibit the expression of the output gene *kanR*. TetR is a protein that acts as a homodimer and has been shown to be useful for inducible transgene expression in both eukaryotes and prokaryotes (Bertram and Hillen 2008; Bertram, Neumann, and Schuster 2022; Asensio‐Calavia et al. 2024). Upon acquiring the tumour KRAS gene, the *tetR* repressor gene was excised from the genome via homologous recombination, thereby activating output gene expression. Additionally, three CRISPR spacer regions were designed to degrade the wild‐type KRAS gene while preserving the KRAS sequence with mutations at the G12 locus. In vitro experiments demonstrated that this biosensor detects DNA sequences from LS174T cells, which contain KRASG12D mutation, but not from RKO cells with wild type KRAS.

In addition to DNA, bacteria can also be engineered to sense metabolites or proteins for tumour detection (Yu et al. 2004; Min et al. 2008; Danino et al. 2015; Panteli et al. 2015). For example, an engineered bacterium *Lactococcus lactis* carries tumour antigen binding proteins on its surface, including binding proteins for epithelial cell adhesion molecule and human epidermal growth factor receptor 2, and expresses red fluorescent proteins for imaging. Once this *L. lactis* comes into contact with cancer cells, its surface marker proteins bind to the corresponding receptors on the cancer cells, enabling recognition. Meanwhile, the red fluorescent protein expressed by *L. lactis* can be used for in vitro and in vivo imaging to monitor its distribution and localization in real time (Plavec et al. 2021).

In addition to the readouts such as antibiotic selection and optical imaging mentioned above, readouts including magnetic resonance imaging (MRI), ultrasound imaging, and positron emission tomography (PET) have also been achieved by engineered bacteria, which have been well summarised (Jian, Xiang, et al. 2024; Jian, Yinhang, et al. 2024). Here, we highlight some of the recent examples. *Escherichia coli* Nissle 1917 (EcN) is a facultative anaerobic organism that proliferates predominantly at the interface between the necrotic and hypoxic regions of the tumour (Li et al. 2019). Following intravenous (i.v.), intraperitoneal (i.p.), and intertumoral (i.t.) injection of EcN, EcN preferentially colonises the tumour, potentially allowing for flexible dosing options to meet specific clinical needs (Liu et al. 2021). Due to this property, EcN has been explored for developing tumour diagnostic methods. For example, EcN naturally expresses thymidine kinase, which allows radiotracers such as 18F‐fluorodeoxyglucose to be phosphorylated and trapped inside the bacterial cells. Upon injection into mice, EcN absorbs and concentrates the radiotracer, resulting in distinct high‐signal areas within tumour tissue on PET scans, thus enabling the visual detection of tumours (Brader et al. 2008). Moreover, EcN has been engineered to overexpress ferritin in the tumour microenvironment, augmenting MRI contrast to enhance tumour imaging and aid early cancer detection (Hill et al. 2011). Optoacoustic imaging (OA), particularly multispectral photoacoustic tomography, is another method that allows high‐resolution and high‐contrast in vivo imaging (Gujrati, Mishra, and Ntziachristos 2017). However, using bacteria for OA faces challenges due to weak acoustic signals and poor photostability of the light absorbers (Brunker et al. 2017). Genetic engineering introducing the tyrosinase gene into *E. coli* enables them to express tyrosinase, catalysing melanin production (Yun et al. 2021). Melanin absorption of light energy generates detectable ultrasonic signals during photoacoustic imaging, facilitating early cancer diagnosis and treatment monitoring. Acoustic reporter genes producing gas vesicles can be engineered into bacteria colonising tumours to monitor tumours via ultrasound imaging (Hurt et al. 2023).

In addition to the detection of in situ tumours, the detection of metastases is also of great importance. Liver metastases are the primary site of metastasis for a number of tumour types, including colorectal, breast, and pancreatic cancers (Schroeder et al. 2012). Studies have shown that oral administration of probiotic strains can lead to selective colonisation of hepatic tumours through blood circulation (Hess et al. 2006). A diagnostic platform, PROP‐Z, based on probiotic EcN, was engineered with an integrated *luxCDABE* cassette (Danino et al. 2015). In a mouse model of colorectal cancer metastasis, oral administration of PROP‐Z resulted in rapid migration through the gastrointestinal tract and specific colonisation of the liver in the presence of metastatic tumours, avoiding healthy organs or fibrotic liver tissue. Bioluminescent imaging using *luxCDABE* cassettes tracked tumour location and metastatic progression.

**FIGURE 2:**
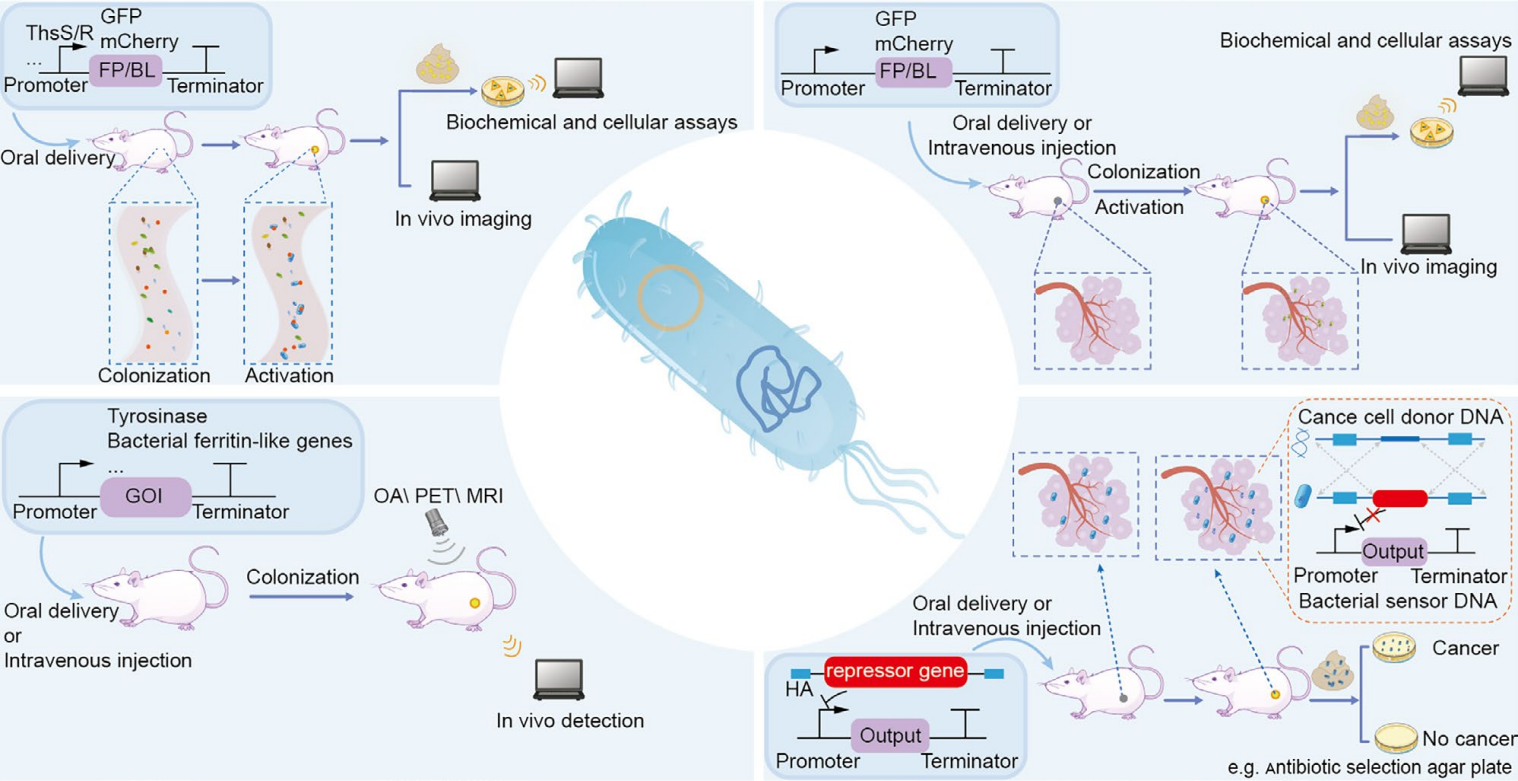
Engineered bacteria offer a range of techniques for disease detection. For tumour detection, fluorescent proteins (FP), and bioluminescence (BL) genes are inserted into bacteria to facilitate in vivo imaging by leveraging the tumour‐targeting properties of certain anaerobic or facultatively anaerobic bacteria or by stool screening for fluorescent or bioluminescent bacteria (Top right). In addition, the antibiotic resistance gene as an output allows bacteria to be screened in vitro on antibiotic plates, which correlates with the presence of tumours (Bottom right) (Cooper et al. 2023). Genes of interest (GOI), such as tyrosinase and bacterial ferritin‐like genes, allow engineered bacteria to produce molecules that respond to imaging modalities, such as optoacoustic imaging (OA) (Yun et al. 2021), positron emission tomography (PET) (Brader et al. 2008), and magnetic resonance imaging (MRI) (Hill et al. 2011), enabling precise tumour localization (Bottom left). For intestinal diseases, engineered microbes are designed to respond to specific disease markers, triggering downstream activation of FP or BL for targeted and specific diagnostic applications (Top left).

Tumour detection using bacteria represents a significant advancement in cancer diagnostics. Genetically engineered bacteria can detect and respond to the unique tumour microenvironment, enabling early detection and precise localization, thereby improving diagnostic accuracy over conventional methods (Figure 2). For instance, tumour‐targeting bacteria equipped with promoters responsive to acidic or hypoxic conditions (Ryan et al. 2009; Flentie et al. 2012; Deyneko et al. 2016; Chien et al. 2022) can sense and adapt to the unique microenvironment of tumours. These bacteria can be engineered to carry reporter genes for imaging, allowing for real‐time visualisation. Future research could involve integrating multiple promoters and reporter genes, such as combining three distinct fluorescent proteins with bioluminescence in a single *E. coli* strain. This multiplexed system (Kusuma et al. 2024), could create a comprehensive diagnostic profile by detecting a range of tumour markers, thus enhancing diagnostic specificity. Additionally, research is needed not only to detect tumours but also to quantify tumour burden, providing precise measurements of tumour size and activity through techniques such as ultrasound imaging (Pflanzer et al. 2014), which can be achieved by the production of gas vesicles by engineered bacteria (Hurt et al. 2023). Furthermore, long‐term monitoring of tumour gene expression is helpful to track tumour progression (Li, Liu, et al. 2020; Li, Yao, et al. 2020; Hurt et al. 2023). Combining bacterial diagnostics with therapeutic delivery systems holds promise for personalised medicine, facilitating targeted treatments monitored and adjusted in real time. Integrating machine learning algorithms to analyse bacterial signals could further improve predictive accuracy regarding tumour behaviour and treatment response, promoting more precise and individualised cancer management strategies.

### 2.2 Bacteria for Oncological Therapy

The potential of bacteria as cancer treatments was first recognised in the 19th century, following observations of tumour regression in patients injected with *Streptococcus pyogenes* and *Serratia marcescens* (Kucerova and Cervinkova 2016). This discovery prompted systematic investigation into the role of bacteria in cancer therapy. Research has demonstrated that bacteria function as natural immune adjuvants, enhancing anti‐tumour immune responses by inducing the secretion of inflammatory mediators such as TNF‐α (Bui et al. 2015), IFN‐γ (Bui et al. 2015), and IL‐12 (Mohamadzadeh et al. 2005). These cytokines stimulate the recruitment and activation of DCs, thereby amplifying the initial innate anti‐tumour response. DCs then migrate to tumour‐draining lymph nodes to present tumour antigens, effectively initiating the activation of anti‐tumour effector T cells (Chandra et al. 2013). For instance, treatment of LM3 adenocarcinoma with *Salmonella typhimurium* strain CVD 915 led to the activation and recruitment of CD4^+^ and CD8^+^ T cells and neutrophils at the tumour site (Vendrell et al. 2011). These recruited cells secreted TNF‐α and IFN‐γ, augmenting the host anti‐tumour immune response.

Although bacterial therapies hold promise, the risk of uncontrolled virulence and proliferation necessitates caution. Bioengineering advancements provide solutions through precise genetic modifications. For instance, VNP20009, a *S. typhimurium* strain with targeted deletion of virulence genes like *msbB* and *purI*, exhibits tumour‐targeting specificity and inhibits tumour growth in murine models (Chen et al. 2018). Additional alterations to attenuated *Salmonella* strains, such as the down‐regulation of endotoxin‐associated genes like *relA* and *spoT*, substantially decrease virulence and enhance safety profiles. These engineered strains stimulate inflammasomes such as NLRP3 and IPAF, prompting the release of pro‐inflammatory cytokines like IL‐1β, IL‐18, and TNF‐α (Phan et al. 2015). Moreover, attenuated *S. typhimurium* has been engineered to express therapeutic molecules, including cytosine deaminase (Mesa‐Pereira et al. 2015), TNF‐α (Yoon et al. 2011), mitomycin C (Pawelek, Low, and Bermudes 2003) herpes simplex virus thymidine kinase (Soghomonyan et al. 2005), and colicin E3 (Leschner and Weiss 2010), thereby augmenting its anti‐tumour efficacy both in vitro and in vivo.

Non‐pathogenic bacteria or probiotics have also been explored in anti‐tumour therapy (Jin et al. 2024). *E. coli* NiCo21 (DE3) engineered with a synchronised lysis circuit regulated by quorum sensing was used as the chassis to express CD47 nanoantagonists (Chowdhury et al. 2019). Upon reaching a population threshold within the tumour, bacterial cells are lysed and release the nanoantagonists, leading to tumour regression and bolstering immune responses in mice. Additionally, employing a hypoxia‐inducible promoter, engineered EcN expressing Tum5 demonstrated anti‐angiogenic effects, suppressing tumour growth and metastasis in melanoma in mice (He et al. 2017). Further modifications, including the expression of the Tum5‐P53 fusion protein, induced apoptosis in liver and cervical cancer cells both in vitro and in vivo (He et al. 2019).

Aside from *E. coli* , a majority of probiotics are lactic acid‐producing bacteria, such as *Lactobacillus*, *Streptococcus*, and *Bifidobacterium*. Probiotics can be beneficial in anti‐tumour therapy as they influence immunoglobulin A production (Singh, Singh, and Gaur 2022; Pei et al. 2024), stimulate DCs activity (Forsythe 2011), and may mitigate the side effects of anti‐tumour treatments (Fuccio et al. 2009). For instance, the therapeutic effects of *Lactobacillus plantarum* on colorectal cancer in mice have been investigated, demonstrating that treatment significantly reduced tumour volumes, prolonged survival, and promoted the migration of CD8^+^ and NK cells to tumour tissues (Hu et al. 2015). Additionally, *L. plantarum* enhanced DC maturation and guided CD4^+^ T cell polarisation toward the Th1 lineage, thereby aiding in the prevention of colon cancer. Modulating gut microbiota composition is also one of the diverse mechanisms that *L. plantarum* uses to exert inhibitory effects on colorectal cancer (Chong 2014). Through genetic engineering, probiotics can be tailored to express anti‐tumour molecules; for example, *Bifidobacterium infantis* expressing soluble fms‐like tyrosine kinase receptor (sFlt‐1), a tyrosine kinase with antiangiogenic properties, effectively suppresses vascular endothelial growth factor (VEGF)‐induced cell proliferation, consequently impeding tumour growth and extending survival in mice murine models (Zhu et al. 2011).

Novel insights into the interplay between microbial metabolites and immunity unveil microbes' pivotal role in shaping immune responses. Oral administration of *Lactobacillus reuteri* generates indole‐3‐lactic acid from tryptophan metabolism within extraintestinal tumours, heightening anti‐tumour immune responses, and enhancing immune checkpoint inhibitor efficacy (Bender et al. 2023). Certain symbiotic flora can infiltrate mammary glands and modulate the microenvironment via the production of metabolites. For example, genera like *Clostridium* induce the production of trimethylamine oxide, which triggers tumour cell apoptosis via PERK activation and boosts CD8^+^ T cell‐mediated anti‐tumour immunity in triple‐negative breast cancer in vivo (Wang, Rong, et al. 2022; Wang, Xu, et al. 2022). Genetically engineered bacteria have been explored to augment cancer immunotherapy by modifying the tumour metabolic milieu. Canale et al. (2021) engineered EcN to convert tumour‐derived ammonia into L‐arginine, a crucial immune regulator. This metabolic shift enhances immune cell activation and infiltration, particularly T cells, within the tumour microenvironment, potentiating anti‐tumour immunity. This approach fosters targeted therapeutic strategies tailored to specific microbial metabolites or metabolic enzymes, offering avenues for optimising immunotherapy by modulating microbial composition or metabolism.

Bacteria present a versatile approach to cancer therapy, offering a dynamic platform for targeted interventions. Engineered bacteria, with their inherent tumour‐homing and genetic adaptability, offer personalised treatment prospects. Progress in synthetic biology enables modification of bacterial strains to respond to the tumour microenvironment. These advanced therapies could feature precise regulatory mechanisms for therapeutic payload release, adaptable to real‐time tumour feedback (Gurbatri et al. 2020). Additionally, integrating bacterial therapies with radiotherapy or chemotherapy may yield synergistic benefits and improved treatment outcomes (Zhang et al. 2023; Wang, Shi, et al. 2024; Wang, Zheng, et al. 2024; Wang, Zhong, et al. 2024). Understanding the complex interactions between bacteria and the host immune system holds promise for enhancing anti‐tumour immune responses and overcoming resistance mechanisms (Liu et al. 2023; Liu, Zhu, and Jiang 2023).

Since different types of tumours are differentially associated with specific microbial communities (Nejman et al. 2020), there have been attempts to isolate microbes directly from tumours and engineer them for diagnostic and therapeutic purposes. Tumour‐resident microorganisms isolated from tumours are expected to have better biocompatibility and targeting capabilities (Goto et al. 2023). Tumour‐isolated *Cutibacterium acnes* has been investigated as a potential tumour‐suppressive agent by leveraging its ability to selectively target and suppress tumour growth (Chintalapati et al. 2024). Future research should focus on how microbes sense different types of tumours through metabolic, immune, or signalling differences in the tumour microenvironment and develop engineered microorganisms that can target different tumours more effectively and specifically. In addition, customised intratumoral microbiota derived from individual cancer patients may have unique anti‐cancer efficacy, which will contribute to the realisation of personalised treatment and improve the precision and effectiveness of cancer treatment.

The spatial organisation of native microbes within tumours is increasingly recognised as an important factor in tumorigenesis and progression (Galeano Niño et al. 2022; Jian, Xiang, et al. 2024; Jian, Yinhang, et al. 2024). However, studies investigating engineered bacteria in this context remain unexplored. Understanding and leveraging that this spatial information could optimise therapeutic efficacy by strategically controlling the distribution of engineered microbes. For example, microbes could be engineered to actively sense and interact with specific cell types or to migrate to targeted regions within tumours via chemotaxis, enhancing immune responses. Additionally, future research could explore how engineered microbes might influence other cell populations, such as immune cells, by improving their spatial distribution within the tumour microenvironment to enhance anti‐tumour activity.

### 2.3 Bacterial Vaccine

The use of live bacterial vaccines in cancer therapy aims to harness attenuated bacteria to trigger the host immune system, inducing a sustained anti‐tumour immune response. Live bacterial vaccines elicit both cellular and humoral immune responses, enhancing host recognition and clearance of tumour antigens (Silva et al. 2014).

BCG, widely used in clinical settings, particularly for high‐grade non‐muscle invasive bladder cancer (Sylvester 2011), exemplifies the role of bacterial vaccines in cancer treatment by stimulating the immune system to enhance cancer cell eradication. Modifying probiotic *L. lactis* to secrete a fusion of Fms‐like tyrosine kinase 3 ligand (Flt3L) and OX40 ligands enables local retention and sustained release of these therapeutics, leading to immune system activation and anti‐tumour immune responses (Zhu et al. 2022). This targeted, low‐toxicity in situ vaccine‐based approach provides long‐term protection against tumour rechallenge in mice. Given the ability of commensal microbes to trigger T‐cell responses (Wegorzewska et al. 2019), efforts have been made to engineer the skin symbiotic strain *Staphylococcus epidermidis* to express melanoma‐associated antigens, aiming to increase melanoma antigen‐specific CD8^+^ T cells and enhance tumour cell killing (Chen et al. 2023). Upon colonisation, the engineered *S. epidermidis* activates tumour‐specific T cells, promoting their circulation and infiltration into both primary and metastatic tumour sites, where they exert targeted cytotoxic effects. It was observed that immune responses to cutaneous colonisers promoted cellular immunity at distal sites and reduced the growth of metastatic melanoma.

Bacteria loaded with nanomaterials have emerged as promising cancer vaccine platforms, leveraging their unique capabilities to biosynthesize nanoparticles and interact with the host immune system (Lu et al. 2023; Niu et al. 2024; Oetiker et al. 2024). For instance, MnO₂‐mineralized bacteria have been shown to activate the host immune response effectively, enhancing tumour recognition and clearance. This strategy not only improves treatment efficacy but also provides long‐term immune protection, preventing tumour recurrence for up to 120 days and delivering effects similar to those of cancer vaccines (Wang, Shi, et al. 2024; Wang, Zheng, et al. 2024; Wang, Zhong, et al. 2024). The mineralization of MnO_2_ reduced the dosage of bacteria required to inhibit tumour growth by alleviating the hypoxic and immune‐suppressive environment in tumours and activating the cGAS‐STING pathway in mice. The tumour microenvironment is typically immunosuppressive so the release of tumour antigens mediated by radiotherapy does not sufficiently activate immune responses. It has been found that injecting *Salmonella* coated with antigen‐adsorbing cationic polymer nanoparticles following radiotherapy resulted in the accumulation of tumour antigens at the tumour's periphery, leading to systemic tumour regression through adaptive immune responses (Wang, Rong, et al. 2022; Wang, Xu, et al. 2022). Using bacteria to transport tumour antigens and activate dendritic cells can be another strategy for in situ cancer vaccination.

Live bacterial vaccines have emerged as a promising strategy in cancer therapy, owing to their capacity to elicit robust and targeted immune responses. However, ensuring their safety remains a critical challenge. A thorough understanding of the behaviour, host interactions, and persistence of these bacteria is essential to mitigate associated risks. Key factors include the assessment of biological properties, genetic stability, and potential toxicities. Rigorous preclinical and clinical evaluations are imperative to identify and address adverse effects, such as systemic toxicity or unintended immune activation. Strategies to optimise vaccine safety, such as bacterial attenuation, genetic containment systems, and the incorporation of suicide genes, offer promising solutions to enhance control without compromising therapeutic efficacy.

Moreover, to achieve large‐scale production of live bacterial vaccines, efficient, stable, and cost‐effective production processes need to be developed. This includes optimising bacterial culture conditions and media to increase growth rates and biomass yields, designing efficient expression systems to achieve high levels of expression and secretion of target proteins and establishing reliable extraction and purification processes to obtain vaccine products with high purity and stable activity (Tripathi 2016).

## 3 Bacteria for Diagnostic and Therapeutic Applications in Intestinal Disease

### 3.1 Bacteria for Intestinal Diseases Diagnostics

Accurate diagnosis of bowel‐related diseases often necessitates invasive and costly procedures, such as endoscopy and biopsy (Shergill et al. 2015). Biomarkers such as specific antibodies, calprotectin, lactoferrin, nitrate, and hydrogen sulfide (Ranjbar et al. 2022; Zou et al. 2024) can be indicative of intestinal diseases generally exhibit short half‐lives or high instability, complicating their detection and leading to suboptimal diagnostic outcomes. For biosensors based on living microorganisms, sensitivity can be optimised through the strategic combination of different genetic elements, including promoters, ribosome binding sites, and terminators. Varying these combinations allows modulation of the output signal strength, enabling adjustment to the biological concentration of the target molecule, which can span from pM to mM ranges. Specificity, on the other hand, mandates that the biosensor accurately detects a specific biomarker without interference from other substances with similar molecular structures that are not disease indicative.

In the context of inflammatory bowel disease (IBD), the production of nitrate via inducible nitric oxide synthase and thiosulfate through inflammation‐induced reactive oxygen species serves as indicative markers of inflammatory processes (Campbell and Colgan 2019). Engineered EcN holds promise as a diagnostic tool for IBD by leveraging these markers. Thiosulfate and tetrathionate can be detected by the thiosulfate sensor (ThsSR) and tetrathionate (TtrSR) molecular sensors, respectively (Daeffler et al. 2017). These systems were integrated into EcN using sfGFP as the readout (Guo et al. 2018). Results demonstrated a positive correlation between fluorescence signals and the concentration of tetrathionate and thiosulfate, suggesting potential utility in detecting intestinal inflammation in mice. An AND logic gate controlled by the two‐component regulatory systems NarX‐NarL and ThsSR was designed to activate the expression of sfGFP in the presence of both nitrate and thiosulfate (Woo et al. 2020). This allows for IBD severity assessment via GFP intensity measurement, while minimising false alarms. In another study, a bacterial biosensor was developed to emit fluorescence in response to NO for the diagnosis of Crohn's disease (McKay et al. 2018). Through a NO‐responsive promoter and GFP gene, bacteria promptly respond to NO level changes, detectable via fluorescence microscopy or flow cytometry.

For diagnostic applications where real‐time monitoring is challenging, the development of storage circuits that can record the history of signal exposure is an alternative strategy. Programmable bacteria have been constructed containing a genetic memory system centered around the cI/Cro region, regulating gene expression in response to specific signals. This enables translation of environmental cues into stable genomic information (Kotula et al. 2014). Utilising CRISPR technology, bacteria can record multiple stimuli, documenting a comprehensive timeline of cellular responses (Sheth et al. 2017). Engineering of CRISPR‐Cas systems also enables bacteria to integrate information from RNA into their genome, facilitating the monitoring of gene expression in response to environmental changes. These scalable sensors offer versatile applications in gut detection, overcoming previous limitations in bacterial biosensors (Tanna, Ramachanderan, and Platt 2021) For example, researchers engineered *Bacteroides thetaiotaomicron* to function as a biosensor capable of recording environmental cues in the gut (Mimee et al. 2015). In the presence of chemical inducers, the bacterium expresses an integrase that can modify a recognition sequence inserted into the genome. The altered genome can then be sequenced to assist in diagnosis based on the events such as chemical exposure.

Nevertheless, the utility of these biosensors continues to hinge on intricate analyses of bacterial protein, RNA, or DNA in faecal samples rather than offering real‐time monitoring from localised bodily regions. A novel approach involves the development of a microcapsule of less than 1.4 cm^3^, which is a pill with an integrated bacterial‐electronic chamber interface in which genetically engineered probiotic biosensors are integrated with custom‐designed photodetectors and readout chips (Inda‐Webb et al. 2023). This capsule exhibits targeted recognition of specific inflammatory biomarkers such as NO, H_2_O_2_, tetrathionate, and thiosulfate via integrated bacterial biosensors. Using the device's low‐power electronic readout circuitry, the luminescence signals from the activated biosensors were relayed to wireless signals that can be transmitted to external devices such as mobile phones for immediate analysis, enabling real‐time visualisation of inflammatory marker concentrations and fluctuations. Notably, the capsule enables uninterrupted operation within the gastrointestinal tract with low power consumption, thereby providing a non‐invasive means for long‐term monitoring of gastrointestinal inflammation.

Additionally, gut microbial components have been investigated to aid the diagnosis of IBD. In a multicenter study analysing stool specimens from healthy humans and patients with Crohn's disease, ulcerative colitis, irritable bowel syndrome, or anorexia nervosa, 16S rRNA sequencing revealed significant dysbiosis in the bacterial flora of Crohn's disease patients (Pascal et al. 2017). The study identified eight specific microbial taxa that could be used for the differential diagnosis of Crohn's disease: *Faecalibacterium*, *Peptostreptococcaceae*, *Anaerostipes*, *Methanobrevibacter*, *Christensenellaceae*, *Collinsella*, *Fusobacterium*, and *Escherichia*. The diagnostic specificity of these microbial markers was 94%, with a sensitivity of 80%. These findings provide a valuable reference for the differential diagnosis of IBD, highlighting the potential of microbiota‐based diagnostics in clinical practice.

While intestinal microbiota markers for IBD can be detected via faecal samples, these approaches face limitations, including variability due to dietary differences and detection delays (Zheng et al. 2024). Recent advances in vitro models of mucus‐adherent gut microbiota have improved the understanding of host–microbe interactions and results suggest that faecal samples fail to fully capture the microecological complexity of the gut mucosa (Calvigioni et al. 2024). Engineered bacteria offer a promising alternative, enabling disease diagnosis through the production of quantifiable markers such as fluorescent proteins or chromogenic substrates. Furthermore, integrating engineered bacterial sensors with ultra low power microelectronics could facilitate in situ detection of gastrointestinal biomarkers critical to health monitoring and disease diagnosis, providing timely and precise results (Inda‐Webb et al. 2023).

Genetically engineered bacterial biosensors hold promise for diagnosing intestinal diseases by detecting specific gut biomarkers, though they also present challenges. Engineered bacteria might interact unpredictably with gut microbiota or escape into the environment, posing potential gene transfer or health risks (Gómez‐Tatay and Hernández‐Andreu 2019; Plavec and Berlec 2020). Synthetic auxotrophy, which limits bacterial survival to the gut environment, or kill switches that trigger bacterial death post‐diagnosis offer potential safeguards (Dang et al. 2023; Armstrong and Isalan 2024). Sensitivity and specificity are additional challenges, as distinguishing among similar gut molecules requires precise engineering. Solutions may include multi‐input biosensors that respond to unique signal combinations or optimised promoter designs (Chiang and Hasty 2023). Additionally, stressors like pH and bile salts impact biosensor functionality; enhanced bacterial stress tolerance and encapsulation methods could improve stability under gut conditions (Rodrigo‐Navarro et al. 2021). There are no clinical trials currently evaluating engineered bacteria specifically for diagnosis, largely due to a lack of clear regulatory guidelines. While safety assessments could mirror those for therapeutic bacteria, regulatory approval would require updated frameworks. Direct use of engineered bacteria for diagnosis may face delays, but packaging them into capsules offers a faster, more feasible pathway, as digital medicines like electronic capsules have already received FDA approval.

### 3.2 Bacteria for Intestinal Diseases Therapy

Microbial communities can influence disease development and prevention by regulating mucosal health and energy metabolism in the gut, thereby modulating host immune activity (Matsuoka and Kanai 2015). Dysbiosis poses risks, leading to diseases like diabetes and inflammatory disorders (Carding et al. 2015). Therapeutic strategies for IBD, particularly using probiotics like EcN, show promise in ameliorating microbiota dysbiosis (Teng et al. 2022). Engineered EcN strains, incorporating the 3‐hydroxybutyric acid (3HB) synthesis pathway regulated by the hypoxic promoter *pfnrS*, exhibit enhanced efficacy in treating intestinal inflammation in mouse models of dextran sulfate sodium‐induced colitis (Yan et al. 2021). This modification enables heightened 3HB production in the anaerobic gut environment, augmenting the growth of *Akkermansia muciniphila* and increasing short‐chain fatty acid production, thereby modulating the gut environment and mitigating intestinal inflammation. Delivery of 3HB using this engineered EcN demonstrates improved efficacy compared to conventional oral 3HB administration. Additionally, the gut bacterium *A. muciniphila* has been extensively investigated for its positive effects on metabolic health, particularly in enhancing gut barrier integrity and exerting anti‐inflammatory actions. Recent studies have provided insights into the mechanisms underlying *Akkermansia*'s therapeutic potential, highlighting its relevance in managing metabolic diseases and obesity‐related disorders (Zhang et al. 2019, 2020). For instance, *Akkermansia* has been shown to positively modulate gut microbiota composition and affect host metabolism by degrading mucin and producing beneficial metabolites, such as short‐chain fatty acids (Zhang et al. 2021). These findings support strategies aimed at enhancing Akkermansia colonisation or activity, including potential applications of engineered bacteria, as promising adjunctive approaches in the treatment of IBD.

Encapsulation of bacteria helps to improve their colonisation and prolong their viability in the host gut (Chen et al. 2024). Chitosan and sodium alginate hydrogels have been employed to encapsulate engineered EcN strains overexpressing catalase and superoxide dismutase to scavenge reactive oxygen species, relieving intestinal inflammation (Zhou et al. 2022). Bacteria encapsulated with chitosan/sodium alginate reduced inflammation and restored colonic epithelial barrier integrity in murine IBD models more effectively compared to their non‐encapsulated counterparts. Moreover, this engineered EcN strain modulated the intestinal microbial community by increasing the abundance of *Lachnospiraceae* and *Odoribacter*, essential for maintaining intestinal homeostasis.

Other probiotics in addition to EcN have also been explored and engineered for the treatment of intestinal diseases. For example, engineered *Bifidobacterium longum* probiotics, loaded with nanozymes capable of removing reactive oxygen species, can modulate immune responses in IBD (Cao et al. 2023). These probiotics enabled targeted delivery and retention of biocompatible artificial enzymes, reducing the expression of inflammatory mediators. The resulting attenuation of inflammation not only promotes probiotic survival but also accelerates the restoration of intestinal barrier function, positively regulating intestinal microecological balance. Validated in both mouse and canine models, this therapeutic approach demonstrates enhanced efficacy compared to conventional clinical medications.

In summary, bacterial engineering represents a pivotal approach for modulating the intestinal microecological balance and addressing intestinal inflammatory conditions through the manipulation of the composition and functionality of the gut microbiota. This modulation, in turn, influences the host immune response. Nonetheless, the challenges of bacterial survival within the intestinal milieu necessitate strategies like encapsulation to enhance their viability. An alternative avenue involves harnessing beneficial microorganisms native to the human microbiota, genetically engineering them, and deploying them for the treatment of intestinal inflammatory diseases or colorectal cancer, ensuring prolonged survival within the gut environment (Russell et al. 2022; Fan et al. 2024). Moreover, the heterogeneity of the intestinal milieu poses a significant challenge. Varied gut regions exhibit distinct physical and chemical attributes, including oxygen levels, pH, and nutrient content, influencing engineered bacteria's activity and expression of genes of interest. Thus, designing adaptable bacteria capable of optimising performance across diverse gut niches remains a major obstacle.

## 4 Bacteria for Diagnostic and Therapeutic Applications in Other Disease

### 4.1 Bacteria for *Vibrio cholerae* Diagnostics and Therapy

Cholera is an acute diarrheal infection that particularly impacts regions with deficient sanitation infrastructure and limited healthcare access (Ali et al. 2015). Its rapid progression to severe dehydration underscores the urgent need for timely surveillance and prompt case identification, aligning with the World Health Organisation's imperative to avert cholera outbreaks. In response, various genetic circuits have been engineered within *E. coli* to detect *V. cholerae* . These circuits leverage *V. cholerae* quorum sensing system proteins, including CqsS, LuxU, and LuxO, to regulate the expression of a GFP reporter in response to *V. cholerae* signalling molecules. This configuration enables modified bacteria to emit green fluorescence in the presence of auto‐inducer signalling molecule CAI‐1 secreted by *V. cholerae* , achieving highly sensitive and specific detection of *V. cholerae* (Holowko et al. 2016).

In addition to *E. coli* , *L. lactis* was also engineered to be a biosensor for *V. cholerae* (Mao et al. 2018). *L. lactis* naturally produces lactic acid, promoting colonisation resistance by lowering gut pH, which discourages *V. cholerae* growth. To detect the *V. cholerae* infection, *L. lactis* was engineered to express a hybrid two‐component receptor that regulates the expression of mCherry in response to cholera autoinducer 1 (CAI‐1). The fluorescence of mCherry is detectable in faecal samples, allowing rapid diagnosis. This engineered probiotic demonstrated efficacy in improving survival under the challenge of *V. cholerae* in murine models.

### 4.2 Bacteria for Periodontitis Diagnosis Diagnostics and Therapy

One cause of periodontal disease is an imbalance in the oral microbiota, with pathogens like *Mogibacteriaceae*, *Ruminococcaceae*, and *Prevotella* colonising the subgingival region and triggering inflammation (Choi et al. 2021). *Lactobacillus rhamnosus* , a probiotic, helps maintain oral health by competitively inhibiting pathogenic bacteria and producing antimicrobial substances like lactic acid and hydrogen peroxide. It also enhances the host's immune response, strengthening the oral mucosa's defences (Ahola et al. 2002). *Streptococcus salivarius* , another oral probiotic, inhibits pathogen growth through amino acid production and forms biofilms that prevent pathogenic adhesion (Burton et al. 2013). These probiotic actions support the concept of using microbial therapeutics to rebalance the oral microbiome and mitigate disease progression.

Given the role of biofilm in periodontitis pathogenesis, leveraging engineered bacteria to disrupt biofilm formation by pathogens and limit associated bacterial activity presents a promising strategy. For instance, in response to biofilm challenges, a lactic acid bacteria‐based approach was developed using engineered *L. plantarum* and *L. rhamnosus* that inhibit *P. aeruginosa* growth and biofilm formation through acidification (Chappell and Nair 2020). In addition, *Bdellovibrio bacteriovorus* has been engineered with ZnO nanorods, resulting in biohybrids capable of penetrating the biofilm and producing reactive oxygen species. These biohybrids effectively remove the plaque biofilm in vivo, alleviate inflammation, and inhibit bone resorption in rat and rabbit periodontitis models (Tang et al. 2022). The capability of detecting inflammatory markers or pathogenic microorganisms in gingival tissues may also be engineered into these bacteria to enable sensitive and early diagnosis of periodontitis.

Beyond biofilm disruption, engineered bacteria could play a diagnostic role by detecting inflammatory markers or specific pathogens in gingival tissues, enabling early and sensitive diagnosis of periodontitis. Advances in biosensor design have led to bacteria that can detect and report the presence of specific molecules associated with inflammation or infection. For example, SCFA is an indicator of inflammation degree related to the progression of periodontal diseases (Dongiovanni et al. 2023). Recent studies have demonstrated the use of engineered bacteria for detection of SCFA such as propionate and butyrate, where these biosensors generate quantifiable fluorescence outputs, underscoring their potential in biomedical diagnostics (Serebrinsky‐Duek et al. 2023). Such engineered biosensors could allow real‐time monitoring of disease progression and response to therapy, providing clinicians with valuable diagnostic tools. Incorporating such multifunctional capabilities in engineered bacteria has the potential to transform periodontal disease management by offering both therapeutic and diagnostic functions in a single platform.

### 4.3 Bacteria for Metabolic Disease Diagnostics and Therapy

In addressing metabolic disorders like diabetes, engineered bacteria present a promising therapeutic frontier. Conventional treatments often entail drawbacks such as side effects and addiction issues (Khursheed et al. 2019). Genetically modified bacteria offer enhanced glucose control and heightened biomarker sensitivity, promising improved diabetes management. For instance, engineered *E. coli* employs synthetic genetic circuits to detect pathological levels of glucose in urine from diabetic patients, providing easy operation and an enhanced signal‐to‐noise ratio (Courbet et al. 2015). Additionally, sustained regulation of blood glucose levels is achieved through oral administration of an engineered EcN strain expressing a CTB‐IGF‐1 heterodimeric protein, which fuses cholera toxin B subunit and insulin‐like growth factor‐1 (Bazi, Jalili, and Hekmatdoost 2013). *Lactobacillus gasseri* has been engineered to secrete glucagon‐like peptide‐1 (GLP‐1), a hormone that promotes insulin production. This engineered strain has demonstrated the ability to induce intestinal epithelial cells to differentiate into functional, glucose‐responsive insulin‐secreting cells. In diabetic mouse models, the oral administration of *L. gasseri* producing GLP‐1 resulted in increased insulin levels, improved glucose tolerance, and significant reductions in hyperglycemia, effectively establishing a self‐sustaining insulin‐producing mechanism within the gut (Duan, Liu, and March 2015). Similarly, the therapeutic effects of EcN expressing GLP‐1 have also been demonstrated in type II diabetes mellitus mouse models (Wang, Shi, et al. 2024; Wang, Zheng, et al. 2024; Wang, Zhong, et al. 2024). Additionally, *L. plantarum* engineered to express GLP‐1 was tested in monkey models showing antidiabetic effects (Luo et al. 2021). These examples underscore the potential of engineered bacteria to provide both real‐time metabolic monitoring and therapeutic regulation of blood glucose.

## 5 Microbial Consortia

The human microbiota is a vast and diverse ecosystem comprising numerous microorganisms, including bacteria, fungi, and viruses (Hugon et al. 2017). These entities inhabit various niches within the human body, such as the gastrointestinal tract, skin, and oral cavity, collectively forming a dynamic microbial environment that intricately interacts with the host. Interactions between these microorganisms and the human immune system are complex and nuanced, significantly influencing immune system development and functionality (Belkaid and Tamoutounour 2016). Furthermore, these interactions have the potential to greatly impact the nature and magnitude of immune responses.

**FIGURE 3:**
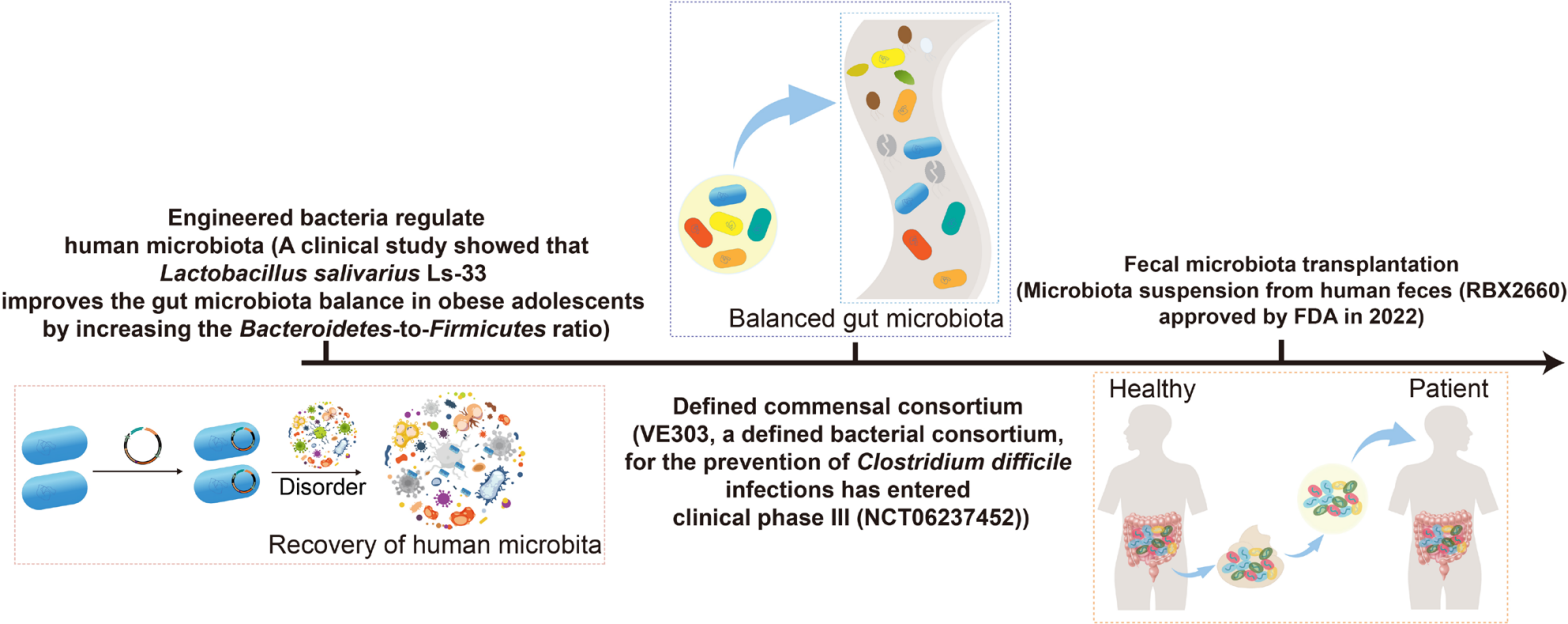
Research processes for utilising and enhancing microbial communities in therapeutic applications. Engineered microorganisms are increasingly utilised to modulate human microbial communities for therapeutic applications. While clinical trials involving single‐engineered strains to modulate microbial communities for disease treatment remain limited, one example is the clinical study of *Lactobacillus salivarius* Ls‐33, which modulates gut microbiota in obese adolescents, enhancing beneficial bacteria and reducing pathogenic taxa (Larsen et al. 2013). Due to the complexity of native microbial ecosystems and the constraints of single‐strain therapies, there is a growing focus on developing defined microbial consortia with specialised roles and interspecies cooperation. An illustrative example is VE303, a defined bacterial consortium that has demonstrated effectiveness in preventing recurrent *Clostridium difficile* infections and is currently undergoing clinical Phase III trials (Louie et al. 2023). However, challenges related to the colonisation of introduced species continue to limit therapeutic success. Faecal microbiota transplantation (FMT), which involves the transfer of an entire gut microbiota from a donor to a recipient, has shown promise in improving colonisation efficiency and enhancing therapeutic outcomes. For instance, RBX2660, an FDA‐approved FMT product, has demonstrated efficacy in reducing recurrent *C. difficile* infections, providing a viable alternative to conventional treatments (Khanna et al. 2022). Notably, FMT holds considerable potential in cancer treatment, providing a robust foundation for enhancing microbial‐based therapeutic strategies.

In the context of tumour immunotherapy, the composition and functionality of the human microbiota have gained considerable recognition (Gharaibeh and Jobin 2019). Understanding the microbiota comprehensively promises valuable insights and guidance for refining and developing more effective disease treatment strategies (Table 2, Figure 3).

**TABLE 2:** Examples of applications involving microbial consortia or single microorganisms used to modify host microbial communities for disease treatment.

Bacteria	Disease model	Mechanism	References
*Enterococcus hirae* and *Barnesiella intestinihominis*	Mice with sarcoma	*E. hirae* increased the intratumoral CD8/Treg ratio, *B. intestinihominis* promoted the infiltration of IFN‐γ‐producing γδT cells in cancer lesions	Daillère et al. (2016)
*Bacteroides* spp. and *Burkholderiales*	Mice with sarcoma, melanoma, and colorectal cancer	Microbiota composition influences IL‐12‐dependent T_H_1 immune response and thus the efficacy of CTLA‐4 blockade	Vétizou et al. (2015)
A consortium of 11 bacterial strains	Mice with breast cancer	The 11 healthy human‐associated bacterial strains co‐induced IFN‐γ^+^ CD8 T cells	Tanoue et al. (2019)
*Lactobacillus paracasei* CNCM I‐4270, *L. rhamnosus* I‐3690 and *Bifidobacterium animalis* subsp. *lactis* I‐2494	Mice with high fat diet‐induced metabolic syndrome	Each strain modulates gut microbiota, attenuating macrophage infiltration into epididymal adipose tissue and improving glucose‐insulin homeostasis	Wang et al. (2014)
*Escherichia coli* Nissle 1917 enigneered to synthesise 3‐hydroxybutyrate (EcNL4)	Mice with IBD	EcNL4 increases the abundance of probiotic bacteria such as *Akkermansia*, *Roseburia*, *Clostridium* subcluster XIVa, and *Ruminococcus*	Yan et al. (2021)
*Bifidobacterium longum* loaded with iron single‐atom catalyst (BL@B‐SA_50_)	Mice with IBD	BL@B‐SA_50_ scavenges reactive oxygen species, alleviates inflammation, increases the abundance of Firmicutes, and decreases the abundance of Proteobacteria	Cao et al. (2023)
*Escherichia coli* Nissle 1917 expressing catalase and superoxide dismutase (EcN‐pE)	Mice with IBD	EcN‐pE increases the abundance of *Lachnospiraceae*_NK4A136 and *Odoribacter* in the intestinal flora, both of which produce butyrate to relieve intestinal inflammation	Zhou et al. (2022)
Faecal microbiota transplantation (FMT)	Mice with IBD	FMT improves immune balance in DSS‐induced colitis by modulating STING‐dependent differentiation and ratios of intestinal and splenic immune cells, including Th17, Th1, Th2, and macrophages	Pu et al. (2024)
FMT	Metastatic melanoma (Human patients)	FMT from immunotherapy‐responsive patients enhances anti‐tumour immunity in non‐responders by modulating gut microbiota, activating STING, and boosting CD8+ T‐cell infiltration, improving PD‐1 inhibitor effectiveness in melanoma	Baruch et al. (2021)

### 5.1 Microbial Consortia for Disease Diagnostics and Therapy

The gut microbiota plays a pivotal role in modulating the host immune system, influencing the tumour microenvironment and affecting the responsiveness and tolerability of immunotherapies (Lu et al. 2022). Furthermore, it regulates the absorption and metabolism of immunotherapeutic agents, thereby impacting their stability and bioavailability (Li, Liu, et al. 2020; Li, Yao, et al. 2020). Understanding and manipulating the composition and function of the gut microbiota is crucial for optimising cancer immunotherapy regimens, enhancing treatment efficacy, and minimising treatment‐related adverse effects. Research has demonstrated a strong correlation between the efficacy of anticancer immunotherapy via cytotoxic T‐lymphocyte‐associated protein 4 (CTLA‐4) blockade and the gut microbiota (Vétizou et al. 2015).

Short‐chain fatty acids (SCFAs) are metabolic products generated by intestinal microbiota during dietary fibre fermentation, mainly comprising acetic acid, propionic acid, and butyric acid. These compounds play a crucial role in maintaining the homeostasis of the gut microbiota. The absence of SCFA‐producing bacteria such as *Bacteroides thetaiotaomicron* and *B. longum* affects intestinal stability and colonisation of pathogens (Wang et al. 2023). SCFAs are also involved in orchestrating anti‐tumour immune responses and strengthening cancer immune surveillance (Kim et al. 2016). Therefore, manipulating gut microbial composition via engineered bacteria to enhance SCFA production presents a promising avenue for therapeutic intervention. For instance, *E. coli* has been genetically modified to sustain 3HB production for colitis management (Yan et al. 2021). After integrating an exogenous 3HB biosynthetic pathway into the *E. coli* genome, the new strain ECNL4 demonstrated sustained 3HB production within the anaerobic environment of the intestinal tract. Experimental results highlighted ECNL4's direct mitigation of intestinal inflammation and indirect modulation of the intestinal milieu by enhancing probiotic abundance, leading to significant SCFA production with therapeutic efficacy. These findings illuminate the intricate interactions between microorganisms for intestinal metabolic homeostasis and underscore the pivotal role of specific microorganisms in maintaining a healthy gut ecosystem, offering new insights into gut microbiota dynamics and microbe‐based therapeutic strategies.

In addition to modulating the microbiota using single engineered strains, microbial consortium can also be used for treatment (Mimee, Citorik, and Lu 2016). For example, a commercially available cocktail of *Bifidobacterium* species, including *B. breve* and *B. longum* , modulates gut microbiota and gut immune cell populations, enhancing antigen‐presenting cells and CD8^+^ T cells, thereby bolstering tumour immunity (Sivan et al. 2015). Application of more complex microbial consortia has been reported as well. A consortium of 11 bacterial strains isolated from healthy human donor faeces was carefully assembled. Oral administration of such symbiotic microbial communities to mice triggers IFN‐γ‐expressing CD8^+^ T cell populations, enhances CD8^+^ T cell functionality, and fosters an anti‐tumour immune environment (Tanoue et al. 2019). Due to the diverse mechanisms by which the microbiota participates in physiology, such a strategy has potential in treating diseases other than tumours, such as infections, inflammation, and metabolic disorders (Charbonneau et al. 2020). For example, the 11‐strain consortia also protected mice from the infection of the pathogen *Listeria* (Tanoue et al. 2019).

For diagnostic purposes, the composition of the gut microbiota serves as a predictive marker for responsiveness to anti‐PD‐1/PD‐L1 therapy in solid tumours (Routy et al. 2018). Patients with a favourable response to immunotherapy exhibit a significantly richer and more diverse gut microbiota. In contrast, non‐responders typically have a simpler and more stable microbiota structure. Recent research revealed that enrichment of the gut microbiota in melanoma patients with *E. faecalis* spp. correlated with a high response to anti‐PD‐L1 therapy, while enrichment with *Bacteroides thetaiotaomicron* and *E. coli* was predominant in non‐responders (Gopalakrishnan et al. 2018). Notably, transplantation of faecal microbiota from responders into germ‐free mice enhanced the therapeutic efficacy of immune checkpoint inhibitors. This enhancement was linked to an increase in intratumoral mature DCs as well as IFNγ^+^ CD8 tumour‐infiltrating lymphocytes (Tanoue et al. 2019).

The application of microbial communities in disease treatment and diagnosis is garnering increased attention. Detailed investigations into microbial communities have elucidated their composition and function in relation to various diseases. These insights have paved the way for innovative diagnostic methodologies and therapeutic strategies. However, point‐of‐care diagnosis based on the microbiota and personalised treatment using microbial consortia remain challenging.

### 5.2 Microbial Consortia Therapy on Comorbid Conditions

Conventionally, a single drug is typically administered to patients to treat a specific type of disease. However, disease progression is multifaceted and often involves comorbidity. Patients can have multiple diseases simultaneously, complicating treatment and affecting therapy outcomes.

Such comorbidity often involves changes in the human microbiota. Dysbiosis of the gut microbiota is linked with chronic conditions such as inflammatory bowel disease, obesity, type 2 diabetes, and autoimmune disorders (Yoo et al. 2013). Similarly, imbalances in oral microbiota are associated with periodontal disease, cardiovascular issues, and arthritis, while skin microbiota imbalances are linked with conditions like eczema and acne (Xu et al. 2023). These findings underscore the significant impact of microbiota on human health and disease, highlighting the importance of restoring normal microbial community structures.

As bacterial therapy functions through diverse mechanisms, it can exhibit additional benefits aside from targeting the primary disease. For example, when using bacteria for anti‐tumour treatment, by modulating the gut microbiota's structure and function, bacteria can alter the host's immune system and overall health, impacting the development and progression of other diseases. Metabolites and immunomodulators produced by these bacteria may confer additional benefits, such as alleviating intestinal inflammation or mitigating symptoms of metabolic disorders (Gasaly, de Vos, and Hermoso 2021). In addition, bacterial consortia alleviate gut inflammation by correcting dysbiosis, activating IL‐10 in immune cells, reducing inflammation, and restoring metabolic profiles. These mechanisms may also target conditions like hepatic encephalopathy, obesity, and type 2 diabetes linked to chronic intestinal inflammation (van der Lelie et al. 2021).

Consortia containing multiple microbes have more diverse and systematic effects than single‐strain therapy and may be a potential strategy for treating comorbidities. Consortia of probiotics have been demonstrated to help regulate intestinal microecological equilibrium, reduce intestinal inflammation, and mitigate metabolic disorders such as metabolic syndrome and obesity. Recent studies have highlighted the regulatory effects of probiotics on gut microbiota in mitigating high‐fat diet‐induced metabolic syndrome in murine models (Wang et al. 2014). Administration of specific probiotic strains, including *Lactobacillus paracasei* , *L. rhamnosus* , and *Bifidobacterium animalis* subsp. *lactis*, has shown their ability to reduce weight gain, enhance glucose‐insulin balance, and alleviate hepatic steatosis in high‐fat diet‐fed mice. These probiotics significantly reduced pro‐inflammatory macrophage infiltration in adipose tissue and decreased inflammatory markers, with notable efficacy in ameliorating systemic inflammation. Other combinations of *Lactobacillus*, such as *L. plantarum* and *Lactobacillus campestris*, have also demonstrated modulation of hepatic lipid metabolism as well as reduction of fat accumulation and associated inflammation (Yoo et al. 2013).

In summary, because the balance of human microbial communities has an extensive influence on physiology, therapy using live bacteria or microbial consortia holds great promise for treating patients with comorbidities. Further investigation is necessary to assess the safety and overall impact of these approaches on health.

## 6 Outlook

Bacteria are emerging as promising tools for therapy and diagnosis, offering many advantages. Primarily, gene editing techniques allow the customised design of bacteria to target specific diseases. Engineered bacteria can be programmed to combat cancer cells, enabling targeted therapies while minimising harm to healthy tissues and reducing unnecessary drug use. Once the strains are constructed, culturing and reproducing bacteria are easy to scale up and cost‐effective, provided the stability of the strain in cultures is monitored and maintained. The development of intelligent, multifunctional genetic circuits in bacterial chassis represents a pivotal advancement toward next‐generation personalised medicine (Liu et al. 2023; Liu, Zhu, and Jiang 2023). For instance, the engineered *E. coli* Nissle strain i‐ROBOT has been designed to sense the intestinal disease marker thiosulfate and subsequently release the immunomodulator AvCystatin, providing targeted therapeutic action (Zou et al. 2023). Similarly, other engineered *E. coli* Nissle strains have been developed to both detect and treat colorectal tumours, exemplifying a dual diagnostic and therapeutic approach (Gurbatri et al. 2024). These innovative platforms highlight the potential of smart bacterial therapies in achieving tailored, responsive treatments for complex diseases.

There have been engineered bacteria approved by FDA for medical application, including Vivotif, an oral typhoid vaccine, and Vaxchora, an oral cholera vaccine. Advances in synthetic biology, such as genome editing and gene expression regulation, have led to robust technologies that show promising outcomes in preclinical studies. However, no engineered bacteria have yet been approved for therapeutic or diagnostic purposes.

A growing number of engineered bacterial strains are progressing thorugh clinical stages for disease treatment; refer to (Riglar and Silver 2018; Li et al. 2021; Gulig et al. 2024) for details. Two notable examples have reached phase III trials. SYNB1934, developed by Synlogic, is an engineered EcN designed for oral administration to treat phenylketonuria. ADXS11‐001, an engineered *L. monocytogenes* , is being tested for the treatment of HPV‐positive cancers. These clinical advances highlight the potential of next‐generation bacterial therapies for addressing challenging diseases.

The use of engineered bacteria in diagnostics and therapy requires careful biosafety assessment, addressing concerns such as uncontrolled colonisation and proliferation, unexpected immune responses, and disruption of the commensal microbiome. Incorporating “self‐destruct” mechanisms, like “suicide switches” that trigger bacterial cell death post‐treatment, offers a promising strategy to mitigate these risks (Din et al. 2016), While these mechanisms aim to minimise adverse effects and reduce microbiome disturbance, extensive clinical validation is still required. Furthermore, understanding bacterial survival dynamics in the human microenvironment, interactions with native microbes, and immune system responses is critical for therapeutic success. Real‐time monitoring of engineered bacteria during treatment could provide significant benefits. For applications where bacterial colonisation is unnecessary, encapsulating engineered bacteria offers a viable solution to mitigate risks. Packaging these bacteria into FDA‐approved digital capsules could potentially facilitate regulatory approval for their clinical use (Mimee et al. 2018; Inda‐Webb et al. 2023).

The colonisation ability of engineered bacteria within the host is critical for achieving therapeutic efficacy and long‐term stability yet remains challenged especially in the complex and variable gut environment. Successful colonisation hinges on both intrinsic biological traits of engineered strains‐such as adhesion, motility, and immune interactions and favourable growth conditions, including nutrient availability, pH, and oxygen levels. Encapsulating engineered strains in biocompatible materials can protect them from gastric acidity and enable targeted intestinal release (Yu et al. 2022). Coating beneficial bacteria with poly(ethylene glycol) has been shown to enhance their ability to penetrate mucus and colonise the gastrointestinal tract (Chen et al. 2024). Additionally, co‐administration with prebiotics and oral delivery systems, like capsules, may further support survival and gut retention (Chua et al. 2020). Further investigation is needed to investigate metabolic engineering and pathway modulation to improve competitive stability within the gut. Systems biology and multi‐omics approaches are promising for understanding interactions among engineered bacteria, the host, and native microbiota, while innovative genetic circuits could provide self‐regulation capabilities to adapt to dynamic host environments, thereby enhancing colonisation efficacy.

Advancements in gene editing technology are leading to significant breakthroughs in disease diagnosis and treatment using non‐model strains. These strains, distinguished by unique physiological and metabolic traits, adapt effectively to specific environments, offering new avenues for therapeutic intervention. While traditional gene editing tools face limitations in these strains, advancements like CRISPR‐Cas technology enable precise gene regulation, elucidating their roles in disease contexts. Diversifying chassis organisms expands the repertoire of biotherapeutic systems, leveraging strains with specialised adaptations like acid and heat tolerance. These resilient bacteria, capable of synthesising and secreting therapeutic molecules, hold promise for innovative disease treatments.

In addition, engineered bacteria offer a promising alternative to traditional antibiotics through mechanisms such as quorum quenching, bacteriocin production, and biofilm disruption. Quorum quenching disrupts bacterial communication systems, preventing coordinated actions like biofilm formation and toxin production (Mukherjee et al. 2018; Li et al. 2023). Bacteriocins, antimicrobial peptides selectively targeting pathogens, spare beneficial microbiota from harm (Mazzolini et al. 2023). Enzymes produced by engineered bacteria degrade biofilm matrices, exposing pathogens to immune defences or other treatments (Eghbalpoor et al. 2024). Additionally, some strains are designed to deliver immune‐activating molecules, bolstering the host's defences (Hackett 2003). These strategies help to prevent antimicrobial resistance (AMR) due to antibiotic misuse.

It is also crucial to ensure that engineered organisms do not transfer antibiotic resistance genes to other members of the resident microbiota through horizontal gene transfer. One way to address this is by removing antibiotic resistance genes used during strain development. Another approach involves biocontainment strategies (Cubillos‐Ruiz et al. 2022). For instance, in a study using engineered *L. lactis* to express β‐lactamase for degrading antibiotics in the gut and preventing antibiotic‐induced dysbiosis, the β‐lactamase gene was split into two separate fragments, which were inserted into different parts of the bacterial genome. The fragments produce protein segments that are secreted and must bind together outside the bacterial cells to form a functional β‐lactamase. This design ensures that β‐lactam resistance cannot be horizontally transferred to other bacteria.

Compared to single‐strain therapy, using microbial consortia composed of naturally occurring organisms with complementary functionalities offers advantages. Native bacteria have better compatibility and stability in the human body, reducing the risk of unintended side effects or immune reactions (Gilbert et al. 2016). Regulatory approval is also simpler for non‐engineered microbes, especially those with GRAS status, facilitating faster clinical application. Additionally, natural consortia lower the risk of horizontal gene transfer, reducing ecological and microbiome‐related concerns. However, challenges remain. Designing stable consortia requires understanding complex interspecies interactions, which become more unpredictable in the human body. Consortia composed of native microbes often lack specific functionalities, such as fluorescent readouts or drug production, that engineered bacteria can deliver with precision. Furthermore, biosafety assessments are not straightforward. For instance, while FDA‐approved faecal transplantation demonstrates the therapeutic potential of microbial consortia, reports of fatal outcomes highlight the complexity of ensuring safety in such applications.

In addition, detailed mechanistic insights are needed to predict the interaction between engineered bacteria and native microbial community. Engineered bacteria, as exogenous entities, may interact with the host microbiota by competing for niches and nutrients or directly modulating the microbial community composition. They can also influence host‐microbe signalling pathways, such as toll‐like receptor pathways, thereby affecting immune responses (Gurbatri, Arpaia, and Danino 2022; Fu et al. 2024). The risk of immune rejection depends on the bacterial species, genetic modifications, and host‐specific factors. To minimise rejection, strategies include engineering bacteria to express immunomodulatory molecules, designing strains compatible with the host microbiota and conducting pre‐screening in diverse microbiome models for safety (Mousavinasab et al. 2023; Hotta, Schrepfer, and Nagy 2024). Additionally, multi‐omics approaches and real‐time microbial monitoring now provide new avenues to track these interactions, revealing how engineered bacteria may affect microbial diversity, nutrient competition, and pathogen suppression within the gut ecosystem (Singh et al. 2023). Design strategies informed by these findings include tailoring bacterial gene circuits to enhance compatibility with specific microbial ecosystems, optimising metabolic outputs to outcompete pathogens, and producing targeted metabolites that regulate immune tolerance (Aggarwal et al. 2023). Such approaches improve therapeutic precision and reduce risks of adverse microbiota disruption. By developing finely tuned bacterial systems that consider these factors, future research could enable safer, more effective therapies that align with the complex dynamics of the host environment, paving the way for precision microbiome engineering in disease management.

Given the uniqueness of each patient's microbiota, therapeutic outcomes are hard to predict, as different microbiomes may respond differently to the same treatment. In the context of personalised medicine, a flexible treatment platform is vital, for example, to tailor to individual microbiome profiles and specific disease phenotypes or to modify engineered bacterial interventions across different microbiota compositions and individual immune responses. This adaptability would enable healthcare providers to modify treatment strategies in real time, ensuring that interventions remain aligned with the evolving dynamics of the patient's microbiome and health status. Currently, aligning advancements in engineered bacterial therapies with personalised medicine has been largely overlooked. Such integration could enhance the efficacy and safety of microbial therapies, improving patient outcomes and advancing microbiome‐based therapeutics. Moreover, the lack of standards and regulatory guidance for using microbial consortia in therapy needs to be addressed to ensure robust quality control and stability. A flexible treatment platform is also necessary to allow for rapid adjustments based on point‐of‐care diagnostics.

In summation, despite several hurdles in disease management and diagnosis, bacterial therapies show significant promise and extensive application potential. With ongoing technological advancements and deepened scientific inquiry, bacteria are poised to play an increasingly pivotal role in medicine, providing more effective and safer therapeutic modalities for the betterment of patient outcomes.

## Author Contributions

**Kai Jin:** conceptualization, visualization, data curation, writing – review and editing, writing – original draft. **Yi Huang:** visualization, data curation, writing – review and editing. **Hailong Che:** writing – review and editing, supervision. **Yihan Wu:** conceptualization, writing – review and editing, project administration, supervision, funding acquisition.

## Conflicts of Interest

The authors declare no conflicts of interest.

## Data Availability

The authors have nothing to report.
